# Lipid-Lowering Therapy in the U.S. Adults Before and After the COVID-19 Pandemic

**DOI:** 10.1016/j.jacadv.2025.101872

**Published:** 2025-06-12

**Authors:** Mohammed Essa, Devesh Malik, Yuan Lu, Huanhuan Yang, Erica S. Spatz, Harlan M. Krumholz, Kamil F. Faridi

**Affiliations:** a Richard A. and Susan F. Smith Center for Outcomes Research, Beth Israel Deaconess Medical Center, Boston, Massachusetts, USA; b Yale University School of Medicine, New Haven, Connecticut, USA; c Section of Cardiovascular Medicine, Department of Medicine, Yale School of Medicine, New Haven, Connecticut, USA; d Center for Outcomes Research and Evaluation, Yale New Haven Health, New Haven, Connecticut, USA

**Keywords:** COVID-19, epidemiology, lipid-lowering therapy, prevention

**Figure:**
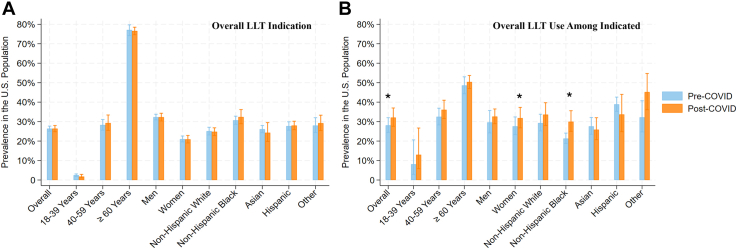

Atherosclerotic cardiovascular disease (ASCVD) remains a leading cause of morbidity and mortality in the United States.^1^ Although professional society guidelines recommend lipid-lowering therapy (LLT) for ASCVD prevention, less than half of eligible adults received treatment before 2020.^2^^,^^3^ Since the onset of the COVID-19 pandemic, cardiovascular mortality rates have also risen,^4^ though LLT use postpandemic remains unknown. To evaluate this further, we assessed LLT indications and use among U.S. adults before and after the onset of the COVID-19 pandemic.

**What is the clinical question being addressed?**

Has the prevalence of LLT indications and use among U.S. adults changed after the onset of the COVID-19 pandemic?

**What is the main finding?**

The proportion of U.S. adults recommended for LLT remained unchanged after COVID-19, but LLT use modestly increased, particularly among women and non-Hispanic Black adults. Overall, LLT uptake remains low, with fewer than 40% of eligible adults receiving therapy.

We included adults ≥18 years in the National Health and Nutrition Examination Survey (NHANES) pre- (January 2017-March 2020) and post-COVID-19 (August 2021-August 2023) survey cycles.^5^ LLT indication was determined by having either existing ASCVD or being aged 40 to 75 years with guideline-based criteria for primary prevention, ie, having diabetes, non–high-density lipoprotein cholesterol level ≥220 mg/dL (alternatively used for low-density lipoprotein cholesterol [LDL-C] ≥190 mg/dL, since LDL-C could not be calculated in the 2021-2023 survey data), or calculated 10-year ASCVD risk ≥7.5% using the pooled cohort equations.^2^ Survey-reported LLT use was defined as taking any medication to lower blood cholesterol. Participants with missing data on LLT use and missing variables for pooled cohort equations, if relevant, were excluded.

Prevalence was determined using survey weights adjusted for combined cycles and accounting for the complex sampling design as recommended by NHANES procedures. Proportions were age-standardized using the 2020 U.S. Census. Prevalence ratios (PRs) were estimated using Poisson regression using the prepandemic cycle as the reference. The study was exempted from review by the Yale Institutional Review Board since all NHANES data are deidentified. Standard errors were estimated using Taylor series linearization, and statistical significance was defined as 2-sided *P* values <0.05. Analyses were conducted using Stata BE version 18 (StataCorp, 2023).

The study included 14,575 participants, representing 241.7 million U.S. adults, in both prepandemic and postpandemic survey cycles. The mean age was 48.5 ± 14.7 years, with 51.8% women, 61.4% non-Hispanic White, 11.3% non-Hispanic Black, 6.1% Asian, and 16.3% Hispanic ethnicity. Overall prevalence of adults with indications for LLT was 26.9% (95% CI: 25.7%-28.0%) prepandemic and remained unchanged postpandemic (27.0%, 95% CI: 25.6%-28.5%; PR: 1.00; 95% CI: 0.93-1.09; *P* = 0.94). The prevalence of adults with indications for LLT remained unchanged after COVID-19 across demographic subgroups (Figure 1).

**Figure 1:**
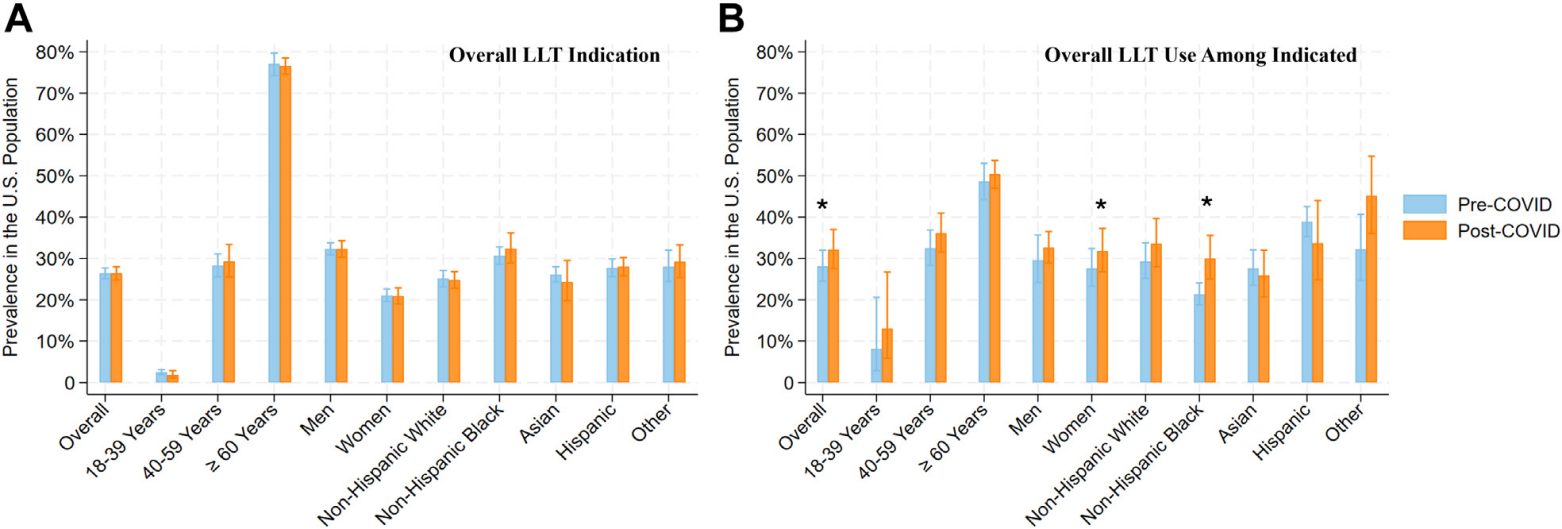
Lipid-Lowering Therapy Indication and Usage Among U.S. Adults Before and After the COVID-19 Pandemic Pre-COVID represents 1/2017 to 3/2020 and post-COVID represents 8/2021 to 8/2023. (A) The prevalence of U.S. adults recommended for LLT based on professional guidelines. (B) The prevalence of LLT use among adults recommended for LLT. Groups marked by an asterisk (∗) had statistically significant changes (*P* < 0.05) based on Poisson regression models adjusted for age, sex, and race or ethnicity. Error bars represent the 95% CI for the estimated proportions. LLT = lipid-lowering therapy.

Among adults recommended to take LLT, utilization of therapy increased from 30.3% (95% CI: 26.0%-35.0%) prepandemic to 35.8% (95% CI: 30.7%-41.2%) postpandemic (PR: 1.09; 95% CI: 1.00-1.18; *P* = 0.041). LLT use among women increased from 29.4% (95% CI: 24.5%-34.8%) prepandemic to 33.8% (95% CI: 28.5%-39.6%) postpandemic (PR: 1.13; 95% CI: 1.02-1.25; *P* = 0.023). LLT use also increased among non-Hispanic Black adults from 23.0% (95% CI: 20.7%-25.6%) prepandemic to 31.9% (95% CI: 27.1%-37.3%) postpandemic (PR: 1.24; 95% CI: 1.04-1.49; *P* = 0.017). There was no significant change in LLT use among other demographic subgroups (Figure 1).

Among adults indicated for LLT without ASCVD, LLT use was 33.5% (95% CI: 29.3%-37.9%) prepandemic and 37.2% (95% CI: 33.2%-41.3%) postpandemic (PR: 1.12; 95% CI: 0.99-1.29; *P* = 0.077). Among adults with ASCVD, LLT use was 39.6% (95% CI: 35.4%-44.1%) prepandemic and 46.3% (95% CI: 37.8%-55.1%) postpandemic (PR: 1.08; 95% CI: 0.98-1.19; *P* = 0.11). There was no significant change in LLT use across demographic subgroups for primary or secondary prevention.

In this analysis, we found that the prevalence of U.S. adults recommended for LLT has remained unchanged since the onset of the COVID-19 pandemic. Therapy use has modestly improved among adults who are recommended for LLT compared to before COVID-19. Increases in LLT utilization were seen for women and non-Hispanic black adults. Greater adherence to guidelines could reflect heightened awareness in addressing disparities, and the recent rise in cardiovascular mortality may thus be unrelated to LLT use. Additionally, the expansion of telemedicine postpandemic may have partially lowered structural barriers to care for women and historically underserved communities.

Despite these encouraging trends, LLT use in the United States remains suboptimal. Less than 40% of eligible adults receive LLT based on guidelines, including less than half of adults with ASCVD. Since inadequate LLT use among the U.S. adults predates the COVID-19 pandemic, more comprehensive strategies are required to close these treatment gaps and reduce ASCVD risk across the population. These strategies should include interventions targeting health care systems and population-based programs as well as patients and clinicians.

Our study is limited to only 1 postpandemic survey cycle, which may reduce accuracy in assessing temporal changes. Data was obtained from adults who agreed to participate, and findings could differ for nonresponders, though survey weights were adjusted for nonresponse. Serum LDL-C was not available in the post-COVID-19 survey cycle, though this would have impacted classification for only a very small number of adults and non–high-density lipoprotein cholesterol was alternatively used. Lastly, our primary prevention population was limited to adults 40 to 75 years of age.

In conclusion, the prevalence of U.S. adults recommended for LLT has remained stable and LLT use has modestly increased since the onset of COVID-19, particularly among women and Black adults. However, LLT use for primary and secondary ASCVD prevention remains inadequate. To reduce cardiovascular morbidity and mortality in the United States, continued efforts are needed to increase LLT utilization based on guideline recommendations.

## Funding support and author disclosures

Dr Lu has received support from the Sentara Research Foundation, the 10.13039/100000050National Heart, Lung, and Blood Institute of the National Institutes of Health (under awards R01HL69954 and R01HL169171), and the Patient-Centered Outcomes Research Institute (under award HM-2022C2-28354) outside of the submitted work. Dr Spatz has received funding from the 10.13039/100000030Centers for Disease Control and Prevention (20042801-Sub01), the 10.13039/100000038U.S. Food and Drug Administration to support projects within the Yale-Mayo Clinic Center of Excellence in Regulatory Science and Innovation (U01FD005938), the 10.13039/100000050National Heart, Lung, and Blood Institute (R01HL151240), and the Patient Centered Outcomes Research Institute (HM-2022C2-28354). Dr Krumholz has received options for Element Science and Identifeye and payments from F-Prime for advisory roles; is a co-founder of and holds equity in Hugo Health, Refactor Health, and ENSIGHT-AI; and is associated with research contracts through Yale University from Janssen, Kenvue, Novartis, and Pfizer. Dr Faridi has received research funding from the National Institutes of Health/National Heart, Lung, and Blood Institute (K23HL161424). All other authors have reported that they have no relationships relevant to the contents of this paper to disclose.
